# Video Watermarking Algorithm Based on NSCT, Pseudo 3D-DCT and NMF

**DOI:** 10.3390/s22134752

**Published:** 2022-06-23

**Authors:** Di Fan, Xiao Zhang, Wenshuo Kang, Huiyuan Zhao, Yingjun Lv

**Affiliations:** 1 College of Electronic and Information Engineering, Shandong University of Science and Technology, Qingdao 266590, China or skd992372@sdust.edu.cn (D.F.); ashley_zx25@163.com (X.Z.); wenshuo99@126.com (W.K.); zhy1021196704@163.com (H.Z.); 2 Department of Electrical Engineering and Information Technology, Shandong University of Science and Technology, Jinan 250031, China

**Keywords:** video watermark, pseudo 3D-DCT, NMF, embedded strength, combined attack

## Abstract

Video watermarking is an important means of video and multimedia copyright protection, but the current watermarking algorithm is difficult to ensure high robustness under various attacks. In this paper, a video watermarking algorithm based on NSCT, pseudo 3D-DCT and NMF has been proposed. Combined with NSCT, 3D-DCT and NMF, the algorithm embeds the encrypted QR code copyright watermark into the NMF base matrix to improve the anti-attack ability of the watermark under the condition of invisibility. The experimental results show that the algorithm ensures the invisibility of the watermark with a high signal-to-noise ratio of the video, and meanwhile has high ability and robustness against common single and combined attacks, such as filtering, noise, compression, shear, rotation and so on. The issue that the video watermarking algorithm has poor resistance to various attacks, especially the shearing attack, has been solved in this paper; thus, it can be used for digital multimedia video copyright protection.

## 1. Introduction

In recent years, the number of online videos has escalated, and it is very easy to publish and obtain video resources. The ensuing digital video copyright issues and data security issues frequently occur, and illegal intruders use modern technical means to arbitrarily access, copy, modify and disseminate media files, seriously threatening the legitimate interests of copyright holders, and even causing harm to personal and property safety [1,2,3]. As an effective method for multimedia copyright protection, video watermarking algorithms with better robustness and more comprehensive attack resistance are urgently needed; a challenging task to combat the current pervasiveness of a large quantity of digital media and tampering tools.

At present, the research of digital watermarking technology for video is mainly based on spatial domain [4,5,6], compression domain [7,8,9,10,11,12] and transform domain [13,14,15,16,17,18,19,20,21]. The principle of the video watermarking algorithm in the spatial domain is to embed watermark data on the basis of a processing pixel value of a video frame image. For example, [4] proposed a fragile watermarking algorithm based on the logic graph, which embeds the watermark into the least significant components of the modified image pixels. However, on the whole, the spatial domain watermarking algorithm has poor anti-attack ability and a narrow application range [5,6]. The video watermarking algorithm based on the compressed domain is a branch of video watermarking, which is usually combined with video coding standards, including video watermarking algorithms based on MPEG-X [7,8], AVC (Advanced Video Coding)/H.264 standard [9,10] and HEVC/H.265 standard [11,12].

Transform domain video watermarking is also an important branch of video experiments, and has a vast range of transform and decomposition methods that can be applied, and that have better robustness. This kind of algorithm is designed to embed and extract the watermark in the transform domain. DWT (Discrete Wavelet Transform) [13], DCT (Discrete Cosine Transform) [14] and SVD (Singular Value Decomposition) [15] are commonly used to transform the image into the transformation domain, which then enables us to embed the watermark in the transform domain. Combining graph-based transformation, singular value decomposition and hyperchaotic encryption, Sharma et al. [16] proposed a video watermarking algorithm, which can solve the address quality loss of data well; however, the algorithm is complex, and the anti-rotation attack performance is poor. Video is a combination of two-dimensional space and one-dimensional time, and three-dimensional transformation is more comprehensive and more suitable than two-dimensional transformation. Fu et al. proposed a watermarking algorithm acting on the 3D-DCT domain. Compared to the algorithm for embedding watermarks on the 2D-DCT coefficients, its robustness is better, but its computational complexity is higher and it takes a longer time [17]. The authors of [18,19] proposed a pseudo 3D-DCT (Pseudo Three-dimensional Cosine Transform) domain video zero-watermarking algorithm; the algorithm performs pseudo 3D-DCT on the moving target centroid block of the keyframe image. The XOR operation of the keyframe eigenvalue sequence is constructed by the AC coefficient, and the encrypted watermark sequence generates a zero watermark, but the anti-rotation attack ability of the algorithm needs to be improved. Li et al. proposed a watermarking algorithm based on DWT and pseudo 3D-DWT, which improved the robustness of the algorithm and the security of the watermark [20]. In addition to the classic transform domain, such as DCT, the DWT and NSCT (Non-Subsampled Contourlet Transform) [21] with multiresolution, and NMF (Non-negative Matrix Factorization) with good robustness against shearing, have also been applied to digital watermarking.

The current transform domain video watermarking algorithm commonly uses 2D transform, whereas the video sequence is a 3D signal; the 3D characteristics of the video frame can be fully utilized to improve the robustness and real-time performance of the algorithm. However, the current video watermarking algorithm based on 3D transform needs a large amount of computation. It is complex, time-consuming, the anti-interference ability is not comprehensive, and the robustness is not strong under certain attacks. A video watermarking algorithm based on pseudo-3D-DCT, NSCT and NMF, which is proposed in this paper, is designed to embed the encrypted watermark into the NMF decomposition of the 3D-DCT of the low frequency component of the NSCT of the keyframe set. The experimental results show that the algorithm combines the advantages of three kinds of changes, and has a good performance in invisibility and robustness. Compared to similar algorithms, this algorithm has more significant advantages in anti-rotation and anti-shearing attacks. On the whole, without increasing the amount of computation, this algorithm takes into account the invisibility and robustness, realizes a more comprehensive anti-jamming effect, and focuses on solving the issues of the poor robustness of most algorithms against shearing and rotation attacks.

In comparison with existing transforms and combinations of transforms, the algorithm suggested in this paper combines the features of three transforms; namely, pseudo 3D-DCT, NSCT and NMF, and is effective in terms of robustness and complete attack resistance. Among these, the usage of pseudo 3D-DCT extracts the video’s temporal information, and the computation is smaller and faster than other transformations, such as pseudo 3D-DWT and 3D-DCT. NSCT is utilized to synthesize the two-dimensional contour features of the frame image in order to improve the resistance of the algorithm to translation and rotation, reduce the number of operations, and enhance the robustness of the algorithm. The dimensionality reduction effect of NMF and the partial perception of the whole are utilized to improve the resistance of the algorithm to shearing attacks. The innovation of this work lies in the effective selection and integration of three transformations as the core of the algorithm, and the performance of the algorithm is tested through numerous experiments, which better achieves the requirements of multimedia high-standard copyright determination with high rapidity, high robustness and wide applicability.

Section 2 focuses on the principles of the three transformations of pseudo 3D-DCT, NSCT and NMF involved in the algorithm. Section 3 illustrates the video watermark embedding algorithm proposed in this paper, containing the algorithm flowcharts, steps and reference examples; in addition, the method of embedding intensity selection and optimization results are given. Section 4 illustrates the video watermark extraction algorithm, giving the algorithm flow, steps and examples as reference. Section 5, experimental results and analysis, shows the anti-attack effect of the algorithm in this paper, and provides a comparison with similar algorithms. From the experimental results, the algorithm in this paper has advantages in terms of the robustness and comprehensiveness of its anti-attack ability. Section 6 summarizes the contents of this paper, and explains the significance of our findings for subsequent research, and the direction of optimization and improvement.

## 2. Related Theories

The algorithm proposed in this paper combines pseudo 3D-DCT, NMF and NSCT to improve the robustness of the video watermarking algorithm. The basic principles of these transformations are as follows.

### 2.1. Pseudo 3D-DCT of Images

DCT is a commonly used transformation in digital watermarking, but most of the images used 2D-DCT to obtain their spectrum. Using 3D-DCT, the video stream can be processed using DCT along the three dimensions (*x*, *y*, *t*) to obtain the spectral distribution of the video in space and time. Fu et al. embedded the watermark into the feature matrix after 3D-DCT, and the experimental data show that the 3D-DCT coefficient is more suitable for watermark embedding than the 2D-DCT coefficient.

As for a video of the size M×N×K (image size is M×N, image frame is K), its 3D-DCT is shown in Equation (1).

(1) $$\begin{array}{l} F(u,v,w)=\frac{\sqrt{8}}{\sqrt{MNK}}c(u)c(v)c(w)\sum_{m=0}^{M-1}\sum_{n=0}^{N-1}\sum_{k=0}^{K-1}f(m,n,k)\\ \;\cos\frac{(2m+1)u\pi }{2M}\cos\frac{(2n+1)v\pi }{2N}\cos\frac{(2k+1)w\pi }{2K}\\ c\left(u\right)=c\left(v\right)=c\left(w\right)=\left\{\begin{array}{ll} 1/\sqrt{2} & u=0,v=0,w=0\\ 1 & \;\mathrm{others}\; \end{array}\right.; \end{array}$$

where u,m=0,1,…,M−1;v,n=0,1,…,N−1; w,k=0,1,…,K−1.

As a matter of fact, the 3D-DCT algorithm has a large complexity and calculation amount. In this paper, pseudo 3D-DCT is adopted to replace 3D-DCT, which greatly reduces the computational complexity and improves the real-time performance of the algorithm. In this paper, the principle of pseudo 3D-DCT is to perform 2D-DCT on the image first. Then, 1D-DCT is performed on the timeline [22]. The transform steps of the pseudo 3D-DCT principle are as follows:

(1) Divide every four keyframes into a group, where each frame is a sub-block of 8×8, and perform a 2D-DCT on each of these blocks.
(2) The DC coefficients of sub-blocks at the same position in each group are connected along the time axis to form a sequence, upon which 1D-DCT is carried out. The result is the pseudo 3D-DCT coefficient.

**Figure 1:**
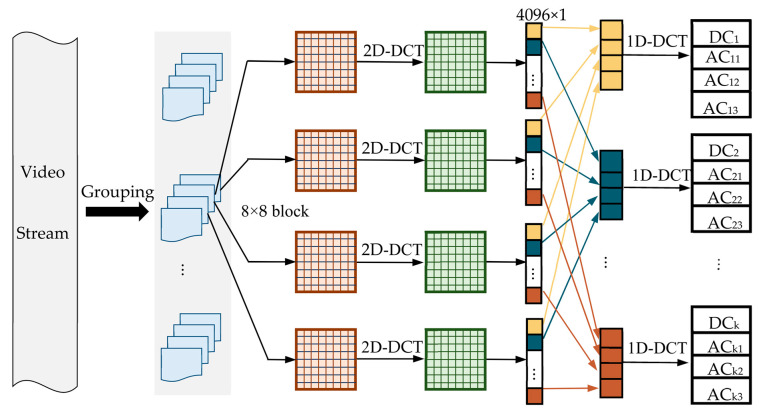
Schematic diagram of the pseudo 3D-DCT principle.

The pseudo 3D-DCT principle is shown in Figure 1. Every four keyframes were divided into groups, and each keyframe was divided into 32 × 32 blocks to obtain 4096 sub-images. 2D-DCT was performed on each sub-image to obtain the DCT spectrum. Four DC coefficients of the DCT results of the sub-image at the same position were arranged in chronological order and 1D-DCT was performed; that is, the pseudo 3D-DCT was completed. Cox et al. [23] and Huang et al. [24] proposed that the embedding region of the watermark should be the most important component of the visual system, namely the low-frequency coefficient. The DC component of the DCT domain is robust and suitable for embedding the watermark, so this algorithm chooses to embed the watermark in the DC component.

### 2.2. NMF Decomposition of Images

NMF is a non-negative matrix factorization proposed by Lee et al. [25] It decomposes any non-negative matrix into two new non-negative matrices by dimensionality reduction. According to NMF, any non-negative matrix *B* of *M* × *N* can be decomposed into the product of the basis matrix *W* and the coefficient matrix *H*, as shown in Equation (2); that is, the column vectors of matrix *B* can be regarded as the sum of all column vectors in *W* multiplied by the corresponding column vectors in *H*.

(2) $$B_{m\times n}=W_{m\times r}H_{r\times n}$$

where r represents the dimension of NMF decomposition, and satisfies $r<mn/\left(m+n\right)$.

The essence of non-negative matrix decomposition is a process of constrained optimization solution [26], and the iterative rule can be used to solve the basis matrix *W* and coefficient matrix *H*. The key to NMF decomposition is the selection of an objective function and an iteration rule [27]. The objective function and iteration rule selected in this paper are shown in Equations (3) and (4), respectively.

(3) $$\min∥B-WH{∥}^{2}\;W,H\geq 0$$

(4) $$H\leftarrow H\frac{W^{T}B}{W^{T}WH}\;W\leftarrow W\frac{BH^{T}}{B^{T}BH}$$

where “*T*” represents the transpose.

When the video is subjected to a malicious shearing attack, the damage to watermark information is irreversible; the dimension reduction effect achieved by NMF, and the feature of partial perception of the whole, can be applied to the watermark technology to improve its ability to resist shearing attack. Furthermore, its non-negative decomposition form and decomposition result has practical and explicable physical significance.

### 2.3. NSCT of Images

**Figure 2:**
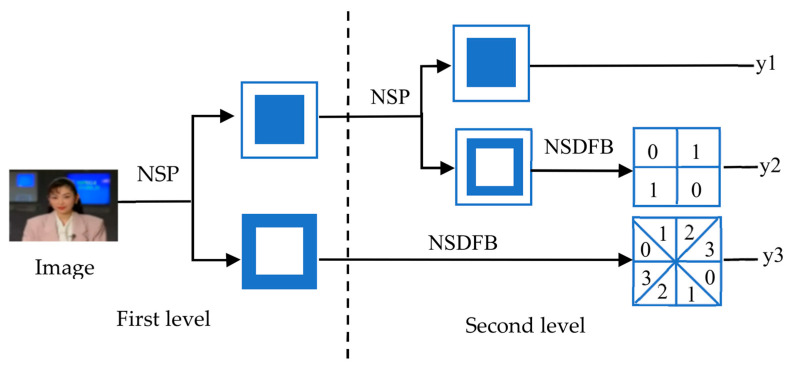
Two-level NSCT decomposition block diagram.

NSCT is a multiscale and multi-decomposition geometric analysis algorithm proposed by da Cunha et al. [28]. It is mainly composed of an NSP (Non-Subsampled Pyramid) and NSDFB (Non-Subsampled Directional Filter Bank) [29]. The two-level NSCT block diagram is shown in Figure 2.

The multiscale characteristics of NSCT are realized by the NSP, and the direction decomposition is based on the NSDFB. The two sets of two-channel filter banks remove upsampling and subsampling from the decomposition process, and the direction sub-bands at all scales are the same size as the original image, and translation invariance is obtained. Applying the NSCT to the digital watermarking algorithm can improve the algorithm’s ability to resist translation and rotation on the one hand, and guarantee the embedding capacity of the watermark on the other hand [30].

## 3. Video Watermark Embedding Algorithm and Embedding Intensity Selection

### 3.1. Video Watermark Embedding Algorithm

**Figure 3:**
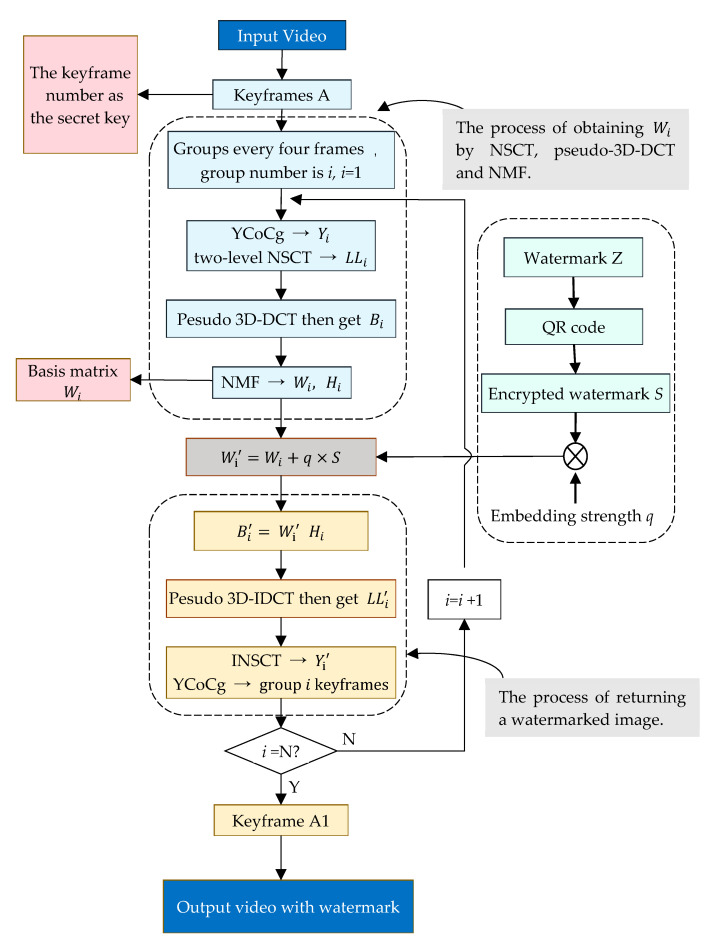
Video watermark embedding algorithm framework flowchart.

The video watermark embedding algorithm framework flowchart based on the pseudo 3D-DCT proposed in this paper is shown in Figure 3. The algorithm combines private copyright information with QR coding technology to generate a QR code watermark, and then scrambles and encrypts the watermark by chaotic mapping to improve the robustness and security of the watermark.

Embedding the watermark on the luminance component Y not only has good robustness, but also has the smallest visual impact [31]; thus, the algorithm in this paper chooses to embed the watermark in the Y component of the image. The embedding algorithm first extracts the keyframes of the video and converts them from RGB to YCoCg color space. The keyframes are grouped in units of four, and the Y components of each group are decomposed by NSCT, pseudo 3D-DCT and NMF in turn, and the watermark is embedded in the base matrix obtained by NMF.

**Figure 4:**
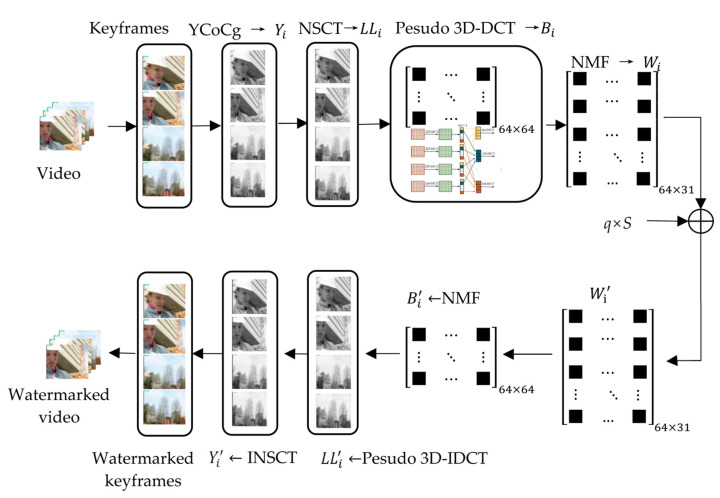
An example of a video watermark embedding algorithm.

Figure 4 is an example of the video watermark embedding algorithm of the video Foreman. Four images are obtained as a keyframes group. Both the process of obtaining $W_{i}\;$ by NSCT, pseudo 3D-DCT and NMF, and the process of returning a watermarked image, are operated. Finally, the watermarked video is produced, and the secret key and basis matrix are saved in the third party.

The process of watermark embedding is as follows:

(1) Extract the keyframe of the video and save the frame number as the key.
(2) Transform the keyframe image into YCoCg color space, and group its Y components into a group every four frames.
(3) Perform two-level NSCT on four Y-component graphs of the group, denoted as $Y_{i}$ = $\left\{Y_{i1},Y_{i2},Y_{i3},Y_{i4}\right\}$, and take their low-frequency sub-bands, denoted as $LL_{i}$ = $\left\{{LL}_{i1},{LL}_{i2},{LL}_{i3},{LL}_{i4}\right\}$, where $LL_{i1},LL_{i2},LL_{i3},LL_{i4}$, respectively, are the low-frequency sub-bands of the two-level NSCT of the four Y-component graphs.
(4) In the pseudo 3D-DCT of ${LL}_{i}$, the DC coefficient matrix with the DC coefficient raised dimension is denoted as $B_{i}$.
(5) NMF with r=31 was performed on the $B_{i}$ matrix of group *i*, and the basis matrix $W_{i}$ was saved. The decomposition error is:
  (5) $$E_{i}=B_{i}-W_{i}H_{i}$$
  where $E_{i}$ is the error matrix; $W_{i}$ and $H_{i}$ are the basis matrix and coefficient matrix after NMF, respectively.
(6) The encrypted watermark *S* is additive embedded into $W_{i}$ to obtain a new basis matrix $W_{i}^{\prime }$. The embedding method is:
  (6) $$W_{i}^{\prime }=W_{i}+qS$$
  where q is the embedding strength of the watermark.
(7) Synthesize $E_{i}$, $W_{i}^{\prime }$, $H_{i}$ into a non-negative matrix:
  (7) $$B_{i}^{\prime }=W_{i}^{\prime }H_{i}+E_{i}$$
(8) Perform inverse pseudo 3D-DCT on the $B_{i}^{\prime }$ to obtain the low-frequency sub-band $LL_{i}^{\prime }$ containing the watermark, and then perform inverse NSCT to obtain the brightness component containing the watermark $Y_{i}^{\prime }$.
(9) $Y_{i}^{\prime }$ is combined with Co and Cg components to obtain a watermarked keyframe image.
(10) Place the keyframe back into the video according to the frame number to obtain the video sequence embedded with a watermark.

### 3.2. Watermark Embedding Strength Choice

The embedding strength of the watermark directly affects the performance of the watermarking algorithm. The robustness of the watermark algorithm will increase with increasing embedding strength, but its invisibility will decrease. The selection of embedding strength should balance the robustness with the invisibility of the watermark.

**Figure 5:**
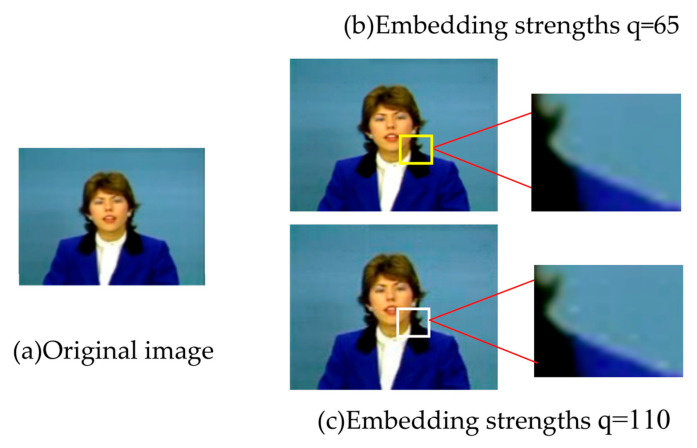
Video images under different embedding strengths.

We conducted multiple experiments on classic test videos (Bus, Claire and Akiyo), and determined the embedding strength of watermarks according to the experimental results. Experimental results show that when the PSNR value of the image containing the watermark is above 40, the invisibility of the watermark is better; that is, the embedded watermark has no obvious influence on the visual effect of the image. In the embedded-strength test experiment, when the value is above 55, the video picture quality will be significantly affected, which may be caused by uneven brightness and spots, etc., and the greater the embedded-strength is, the more obvious the spots will be. Figure 5b,c are the pictures of the Claire video when the imprint embedding strength is 65 and 110 respectively. It can be seen from the partial enlargement that bright spots appear on the background beside the host’s shoulder.

**Figure 6:**
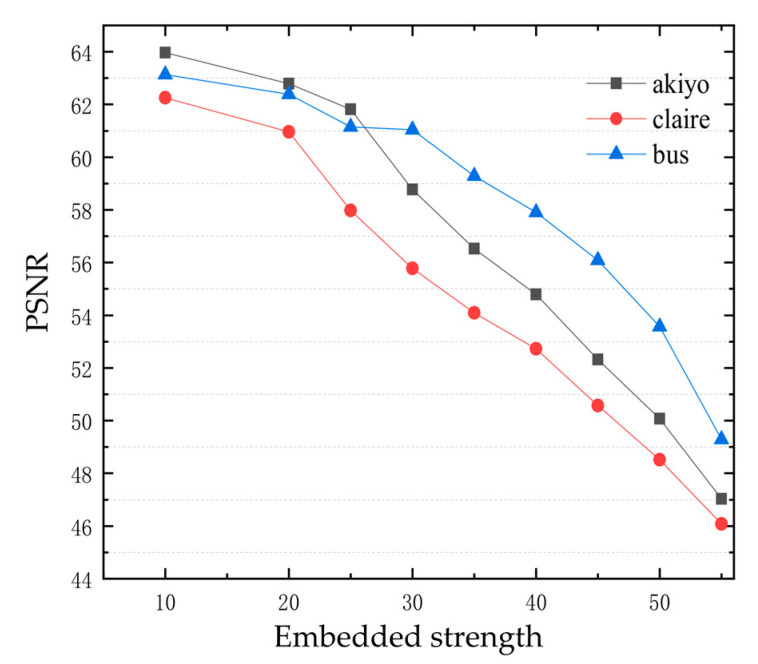
Relationship between PSNR and embedded strength.

**Figure 7:**
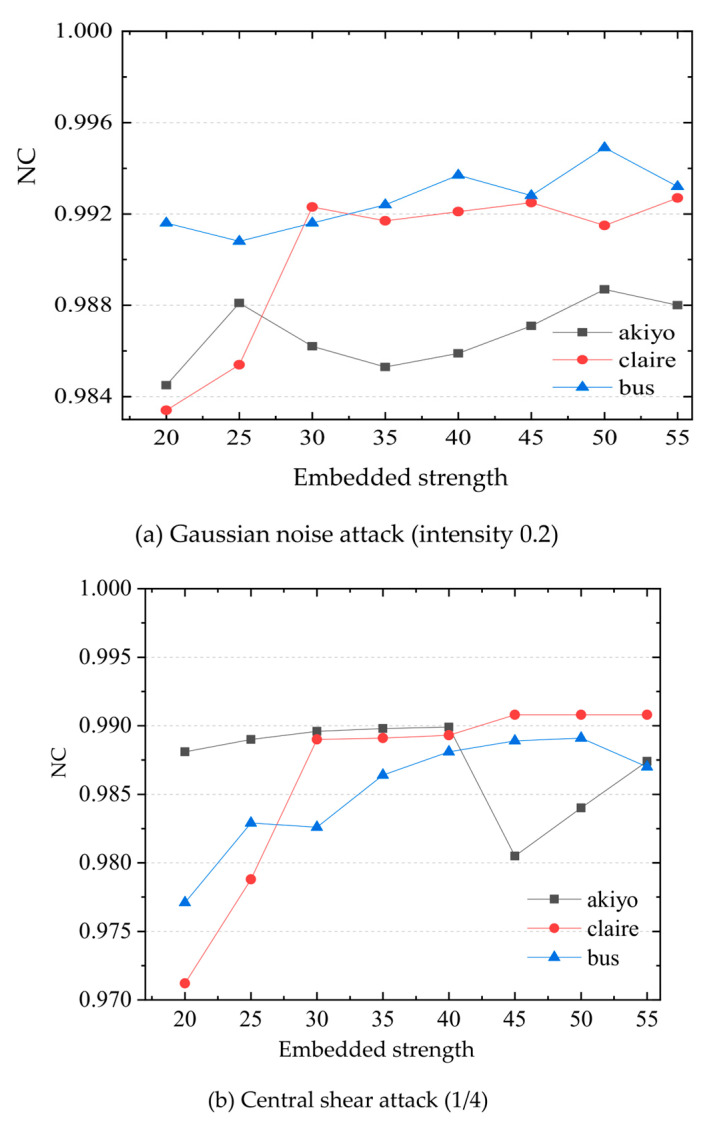

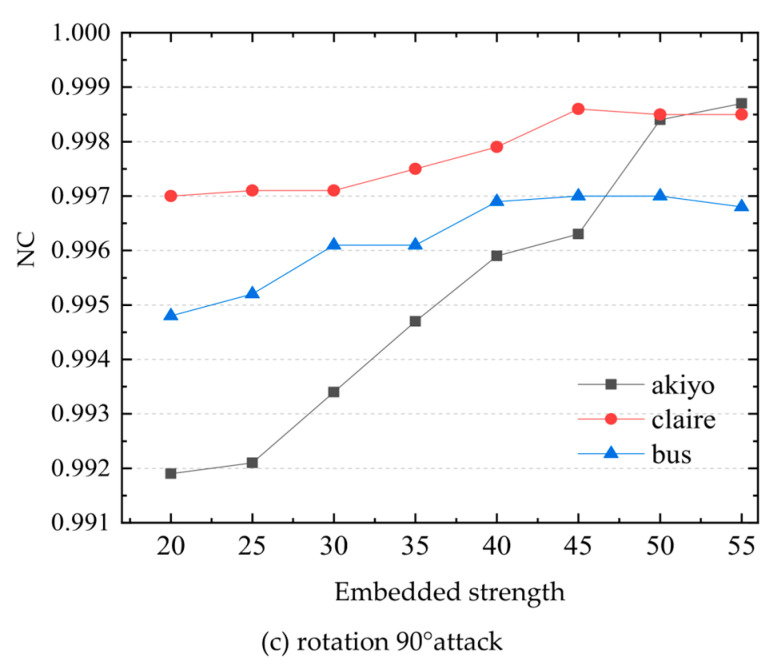
Relationship between watermark NC values and embedding strength under different attacks.

We further quantitatively evaluate the influence of embedding intensity on image quality. Figure 6 shows the PSNR value of images under different embedding intensity. It can be seen that when the embedding intensity is between 10–55, the PSNR values of the images are all higher than 40, indicating good image quality. Therefore, we examine the changes of watermark robustness in the range of 10–55, and determine the final embedding strength value. Generally, the robustness of video watermarking to shear, rotation and noise attacks is poor. This paper focuses on the robustness of experimental algorithms for these three attacks. Under the attack of Gaussian noise 0.2, central shear 1/4 and rotation 90°, the relationship between watermark embedding strength and watermark extraction NC values are shown in Figure 7. On the whole, the NC values increase with the increase in watermark embedding strength, but the video picture quality decreases with the increase in embedding strength. In combination with the embedding strength, PSNR and robustness relationship, the embedding strength is determined as 50.

## 4. Video Watermark Extraction Algorithms

**Figure 8:**
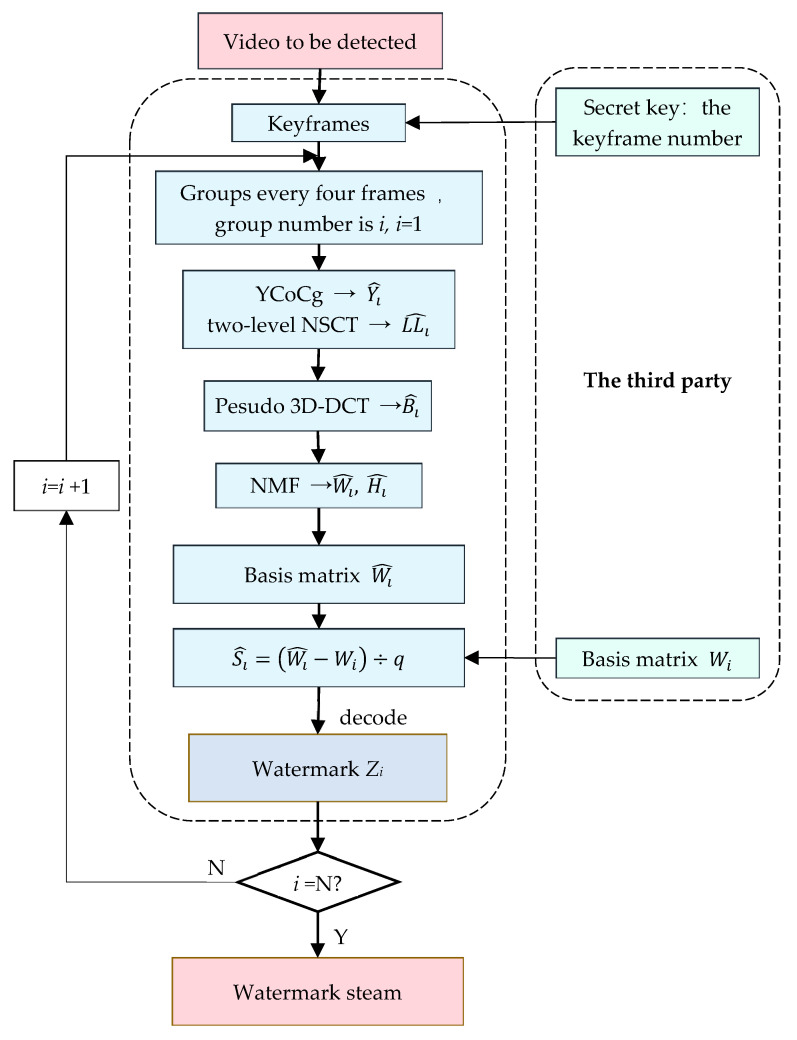
Video watermark extraction flowchart.

Video watermark extraction algorithm is the inverse process of watermark embedding, and its process is shown in Figure 8. The specific steps of the algorithm are as follows:

(1) Find the video keyframe according to the frame number saved by the key.
(2) According to the video watermark embedding algorithm, the NMF of the ${\hat{B}}_{i}$ of group *i* is decomposed into:
  (8) $${\hat{B}}_{i}={\hat{W}}_{i}{\hat{H}}_{i}$$
  where ${\hat{W}}_{i}$ and ${\hat{H}}_{i}$ are the basis matrix and coefficient matrix of ${\hat{B}}_{i}$, respectively.
(3) Using the saved base matrix $W_{i}$, extract watermark ${\hat{W}}_{i}$ from $\hat{S}$ as:
  (9) $$\hat{S}=({\hat{W}}_{i}-W_{i})/q$$
(4) Decrypt $\hat{S}$ to obtain copyright watermark information $\hat{s}$, which can be used for copyright authentication.

**Figure 9:**
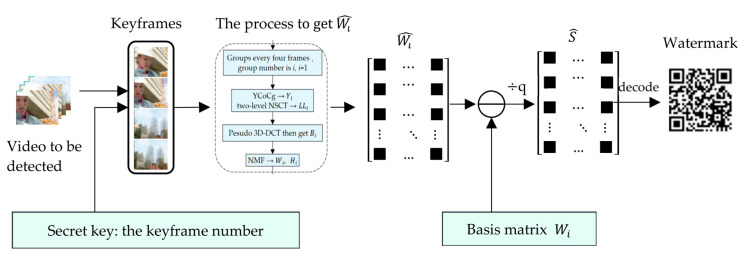
An example of video watermark extraction.

Figure 9 is an example of the video watermark extraction algorithm. Using a secret key, keyframes are obtained from the video. Through the process of obtaining $W_{i}\;$ by NSCT, pseudo 3D-DCT and NMF, one obtains matrix $\hat{W_{i}{}_{\;}}$. Via the basis matrix, which is saved in the third party, the watermark is extracted.

## 5. Experimental Results and Analysis

The experiments in this paper were carried out on the platform of Matlab 2017b, and the classic test videos (Foreman, Claire, Akiyo, Bus) were used as the experimental videos. The video frame size of Foreman, Akiyo and Bus is 352 × 288, and the frame rate is 29 fps; the frame size of Claire is 176 × 144, and the frame rate is 29 fps. Except for the Bus video, which is 5 s, the other three videos are all 10 s. In order to unify the period, the size of the video frame was first redefined as 512 × 512 in the experiment, and the original size was restored after the watermark embedding was completed. The experiment used ‘Shandong University of Science and Technology’ as copyright protection to generate QR code watermark. The watermark size is 64 × 31, the parameters of logistic chaotic encryption in the experiment are $x_{0}=0.1$, μ=4, and the watermark embedding strength value is 50. The robustness and invisibility of the algorithm were evaluated by the PSNR value and the NC value.

### 5.1. Invisibility Experiment Results and Analysis

Four classical test videos were embedded and extracted with watermarks. The number of keyframes extracted by the algorithm and CPU running time are shown in Table 1.

Table 2 shows the PSNR value of the four experimental videos after embedding the watermark and the NC value of the extracted watermark. It can be seen from the experimental results that when the video is not attacked, the PSNR values of the watermarked keyframe images are all close to 50, indicating that the algorithm in this paper has good invisibility; alternatively, the NC value of the extracted watermark is 1, indicating that, using the algorithm in this paper, the resulting watermark can be used for copyright protection.

### 5.2. Robustness of Experimental Results and Analysis

We used the video Akiyo as the test video, and carried out common attacks, such as Gaussian noise, salt and pepper noise, shearing, scaling, rotation, JPEG compression, Gaussian filtering, MPEG4 compression, H.264 compression, etc., and robustness tests under various combined attacks, the results of which are shown in Table 3. From the experimental data, in the case of being attacked, the NC values of the extracted watermarks are all above 0.97, indicating that the algorithm has good robustness.

**Figure 10:**
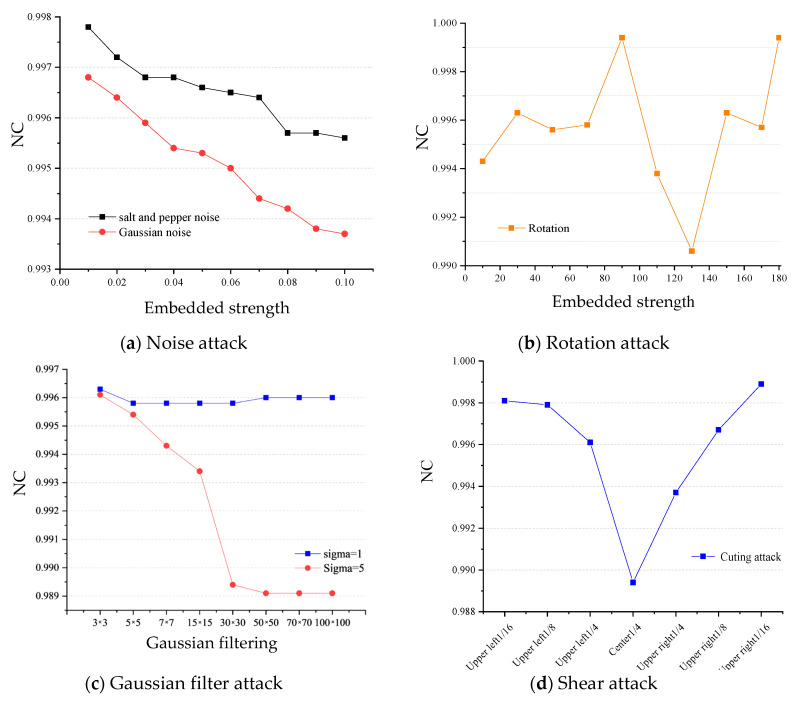
NC values of watermarks under noise, rotation, shear, filter attacks.

In order to further investigate the performance of the algorithm under attack, this paper selected noise, rotation, shearing, filtering and combined attacks to conduct a large-scale experiment, the results of which are shown in Figure 10.

**Figure 11:**
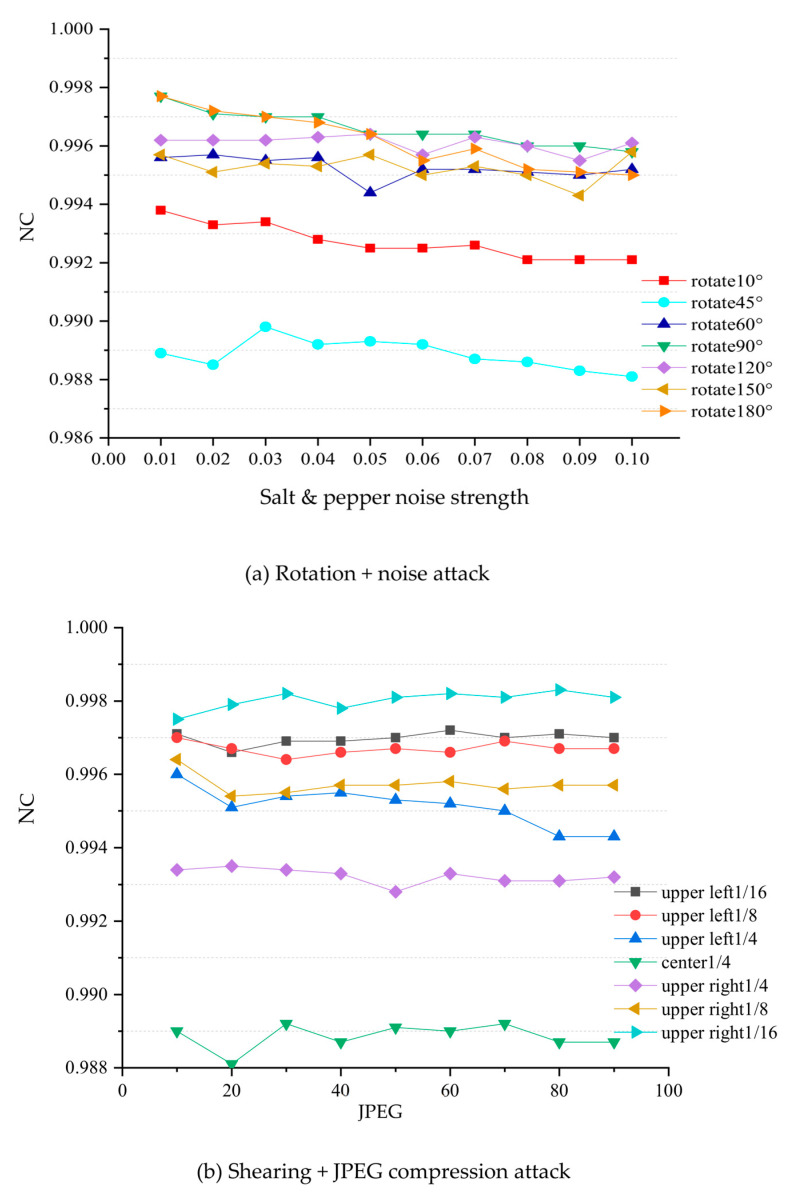

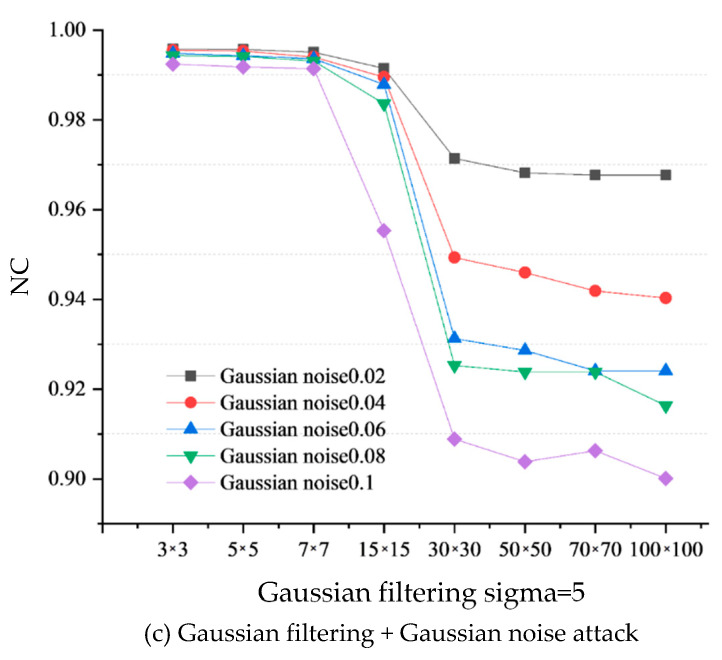
NC values under different combined attacks.

(1) Noise attack. The added attack intensity is Gaussian and salt and pepper noise in the range of 0–0.1. The experimental results are shown in Figure 10a. It can be seen from the results in the figure that, under the noise attack, the NC values of the watermark extracted from the video keyframes are above 0.99, indicating that the algorithm has strong anti-noise ability, especially under the salt and pepper noise attack, for which the NC values are above 0.995. To a certain extent, it can be shown that the resistance of the algorithm to salt and pepper noise is better than to Gaussian noise.
(2) Rotation attack. The added attack is a rotation of 15° in the range of 10–180°, and the result is shown in Figure 10b. It can be seen from the figure that even under a large rotation attack, the NC value of most of the watermarks can still be maintained at about 0.98, indicating that the algorithm has good robustness to rotation attacks.
(3) Shearing attack. The added attack is to cut 1/16, 1/8 and 1/4 in the upper left and upper right corners, and 1/4 in the center. The experimental results are shown in Figure 10c. The experimental results show that, due to the characteristics of the pseudo 3D-DCT and NMF algorithms, the algorithm also shows good robustness to shearing attacks.
(4) Filter attack. The added attack is Gaussian filtering with different window sizes and scales of Sigma = 1 and Sigma = 5. The experimental results are shown in Figure 10d. It can be seen from the figure that the mean values of the NC extracted from the watermark are above 0.98, and the algorithm has strong robustness to filtering attacks under various window sizes.
(5) Combination attack. In this paper, three combined attacks of rotation plus salt and pepper noise, JPEG compression plus cropping, and Gaussian filtering and Gaussian noise under different windows were selected for experiments, and the results are shown in Figure 11. From the experimental results, for the first combined attack, most of the watermark NC values extracted by the algorithm in this paper are above 0.90, which has a good anti-attack ability for the combined attack, and the algorithm is more sensitive to rotation attacks than salt and pepper noise. For the second combined attack, the algorithm in this paper has strong robustness under small-scale cropping and JPEG compression attacks, the extracted watermark NC values can reach more than 0.98, and the sensitivity to cropping attacks is higher than that of JPEG compression. For the third combined attack, the NC values of the watermark extracted by the algorithm in this paper under different window Gaussian filtering and Gaussian noise attacks are all above 0.90, which indicates good resistance, and that it is sensitive to both Gaussian filtering and Gaussian noise attack.

### 5.3. Comparative Experimental Analysis

The algorithm in this paper is compared with two similar algorithms in the literature [32,33]. The algorithm in [32] is a video watermarking algorithm based on the low-frequency sub-band of the Contourlet domain, which performs 1D-DCT on the time dimension for the low-frequency part. For the study of [33], the researchers chose DC coefficients and 2D-DWT low-frequency LL sub-bands for watermark embedding. The watermarks used in the two methods are 32 × 32 binary images, and the algorithm used in the two papers are a binary watermark based on QR coding.

In this paper, the same attack test was carried out on the literature-derived algorithms together with the proposed algorithm. Table 4 lists the experimental results of the Foreman and Bus videos. The table shows that the NC values of the watermark extracted by this algorithm are above 0.9, and the robustness of the proposed algorithm against attacks is better than that of the comparative algorithms. Compared with the literature-derived algorithms, the robustness of the proposed algorithm under shearing, rotation and scaling attack is better, especially for rotation and shearing attacks.

## 6. Conclusions

The video watermarking algorithm presented in this paper combines the advantages of NSCT, pseudo 3D-DCT and NMF to achieve both invisibility and robustness, and can be used in video copyright protection. It can be seen from the experimental results that the algorithm in this research successfully achieves real-time performance and invisibility of video watermarking, and shows high robustness against various attacks, such as noise, shearing, filtering, JPEG compression, etc. Under all kinds of attacks in the experiment, the average NC values of the extracted watermark were above 0.90. Compared with the algorithm using 3D-DCT, the proposed algorithm has high real-time performance and strong anti-attack ability, especially in anti-rotation and anti-shearing attacks. Under rotation attacks in the range of 0–180°, the average NC values were about 0.98; under shearing attacks, the average NC values were above 0.9. In addition, the anti-attack effect under the other attacks also exhibited outstanding performance.

The algorithm presented in this paper improves upon existing approaches in terms of robustness and comprehensive attack resistance. On the one hand, the algorithm presented in this paper will serve as a copyright authentication basis for multimedia that is vulnerable to most single or combined attacks, providing high robustness and comprehensive copyright protection for media publishers and organizations, as well as serving as a credential source for authentication agencies to certify high trustworthiness. The ideas and innovation of this algorithm, on the other hand, have implications for future research, and can be utilized as a useful research direction for high-performance copyright protection algorithms that can be further developed and optimized on this basis.

It should be noted that, with the development of society, researchers will have higher requirements for the real-time performance, robustness, and invisibility of the algorithm. Consequently, in future applications, there is a need to further improve the real-time and robustness of the algorithm. An optional solution is to optimize the NMF and pseudo 3D-DCT algorithms. Beyond that, this algorithm is conditional for saving the secret key and the basis matrix so that the algorithm can be improved in the future, to realize the blinding or semi-blinding of the algorithm. Furthermore, the proposed algorithm is based on the original video in an uncompressed format, but in some application scenarios, the video needs to be compressed for storage and transmission. Therefore, the study of the video watermarking algorithm, combined with the encoding format, is a research direction that needs attention.

## Figures and Tables

**Table 1:** Watermark embedding extraction time of four classical videos.

Video Name	Video Length (s)	Number of Keyframes Embedded with Watermarks	CPU Time (s)
Akiyo	10	12	<75
Bus	5	16	<96
Claire	10	28	<103
Foreman	10	4	<25

**Table 2:** Invisibility experiment results without attack.

Video Name	Foreman	Claire	Akiyo	Bus
Some video frames with watermark	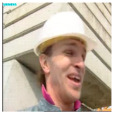	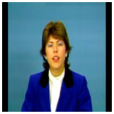	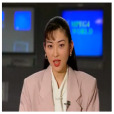	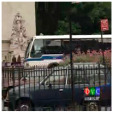
PSNR	47.1679	48.1352	48.0193	49.0118
NC	1.0000	1.0000	1.0000	1.0000

**Table 3:** Robustness experimental results of the algorithm.

Attack Type	Attack Parameter	PSNR	NC	Attack Type	Attack Parameter	PSNR	NC
Gaussian noise	0.01	20.4067	0.9968	rotation	10°	13.5621	0.9943
	0.05	14.2519	0.9950		20°	11.1896	0.9939
Salt and pepper noise	0.01	24.7449	0.9978		45°	8.9730	0.9871
	0.05	17.7592	0.9966	JPEG compression	70	40.4686	0.9989
Shearing	Upper left shear 1/3	7.1723	0.9921		30	36.8852	0.9984
	Down shear 1/3	10.0480	0.9732		5	26.7172	0.9942
Scaling	1/2	40.3844	0.9987	Combined attack	JPEG10 + scaling 1/2	31.9584	0.9973
	2	47.9981	0.9996		Upper left shear 1/16 + Gaussian noise 0.02	11.3136	0.9945
Gaussian filtering	3 × 3	38.5250	0.9965		JPEG10 + salt and pepper noise 0.1	17.3147	0.9936
	7 × 7	36.5973	0.9958		Median filtering + center shear 1/4	9.4794	0.9881
Recompression	Mpeg4	40.978	0.9995		Gaussian filtering 3 × 3 + Mpeg4 compression	13.6111	0.9902
	H.264	39.5714	0.999		Scaling 2 + H.264 compression	20.4327	0.9923

**Table 4:** Comparison of experimental results (NC value).

Experiment Video	Attack Type	Algorithm [32] Algorithm	Algorithm [33] Algorithm	Proposed Algorithm
Foreman	Rotation (10°)	0.8226	0.8209	0.9910
	Rotation (30°)	0.8591	0.8096	0.9941
	Rotation (45°)	0.8330	0.7992	0.9884
	Scaling (1/2)	0.9757	0.9290	0.9992
	Scaling (2)	0.6348	0.9041	0.9995
	Rotation (10°) + Scaling (2)	0.8591	0.8042	0.9803
	Rotation (30°) + Scaling (1/2)	0.8435	0.7924	0.9909
	Shearing (1/8)	0.9078	0.5292	0.9953
	Scaling (1/4)	0.8070	0.3936	0.9913
	Scaling (1/2)	0.6000	0.3235	0.9309
	Median filtering	0.9965	0.9295	0.9972
Bus	Rotation (10°)	0.8887	0.8562	0.9912
	Rotation (30°)	0.7861	0.8208	0.9911
	Rotation (45°)	0.7078	0.7961	0.9871
	Scaling (1/2)	0.9843	0.9473	0.9991
	Scaling (2)	1	0.9138	0.9996
	Rotation (10°) + Scaling (2)	0.8904	0.8279	0.9817
	Rotation (30°) + Scaling (1/2)	0.7809	0.7947	0.9851
	Shearing (1/8)	0.9061	0.5945	0.9973
	Shearing (1/4)	0.8157	0.4789	0.9894
	Shearing (1/2)	0.5965	0.4560	0.9477
	Median filtering	0.9826	0.9469	0.9965

## Data Availability

The data used to support the findings of this study are available from the corresponding author upon request.

## References

[1] Agarwal H., Husain F. (2021). Development of payload capacity enhanced robust video watermarking scheme based on symmetry of circle using lifting wavelet transform and SURF. J. Inf. Secur. Appl..

[2] Sun J., Jiang X., Liu J., Zhang F., Li C. (2021). An Anti-Recompression Video Watermarking Algorithm in Bitstream Domain. Tsinghua Sci. Technol..

[3] Xuecheng S., Zheming L., Zhe W., Yongliang L. (2021). A geometrically robust multi-bit video watermarking algorithm based on 2-D DFT. Multimed. Tools Appl..

[4] Sahu A.K. (2021). A logistic map based blind and fragile watermarking for tamper detection and localization in images. J. Ambient. Intell. Humaniz. Comput..

[5] Munir R. A Secure Fragile Video Watermarking Algorithm for Content Authentication Based on Arnold Cat Map. Proceedings of the 4th International Conference on Information Technology (InCIT), IEEE.

[6] Arab F., Zamani M., Poger S., Manigault C., Yu S. A Framework to Evaluate the Performance of Video Watermarking Techniques. Proceedings of the 2nd International Conference on Information and Computer Technologies (ICICT).

[7] Ahuja R., Singh Bedi S. (2019). Video watermarking scheme based on IDR frames using MPEG-2 structure. Int. J. Inf. Comput. Secur..

[8] Ahuja R., Sharma M., Haque M.J. A Compressed domain Based Robust and Imperceptible Digital Video Watermarking Scheme. Proceedings of the Sixth International Conference on Parallel, Distributed and Grid Computing (PDGC).

[9] Sun Y., Wang J., Huang H., Chen Q. (2020). Research on scalable video watermarking algorithm based on H.264 compressed domain. Opt. Int. J. Light Electron Opt..

[10] Li C., Yang Y., Liu K., Tian L. (2020). A Semi-Fragile Video Watermarking Algorithm Based on H.264/AVC. Wirel. Commun. Mob. Comput..

[11] Dhevanandhini G., Yamuna G. (2021). An effective and secure video watermarking using hybrid technique. Multimed. Syst..

[12] Gaj S., Sur A., Bora P.K. (2020). Prediction mode based H. 265/HEVC video watermarking resisting re-compression attack. Multimed. Tools Appl..

[13] Darabkh K.A., Al-Sheikh R.M., Haddad R.F., Khalifeh A.F. Scene Change Based Video Watermarking Algorithm. Proceedings of the International Conference on Innovation and Intelligence for Informatics, Computing and Technologies (3ICT).

[14] Sun W., Zhao H., Zhang X., Sun Y., Liu X., Lv X., Fan D. (2022). Zero-watermarking Algorithm for Audio and Video Matching Verification. Optim. Algorithms Dyn. Syst..

[15] Fan D., Li Y., Gao S., Wang G., Chi C., LV C. (2020). A Novel Zero Watermark Optimization Algorithm Based on Gabor Transform and Discrete Cosine Transform. Concurr. Comput. Pract. Exp..

[16] Sharma C., Amandeep B., Sobti R., Kumar Lohani T., Shabaz M. (2021). A secured frame selection based video watermarking technique to address quality loss of data: Combining graph based transform, singular valued decomposition, and hyperchaotic encryption. Secur. Commun. Netw..

[17] Fu Y. Robust image watermarking scheme based on 3D-DCT. 2009 Sixth International Conference on Fuzzy Systems and Knowledge Discovery, IEEE.

[18] Yiming L., Chongxiong Z. (2019). Video zero-watermarking algorithm in pseudo 3D-DCT domain. Electron. Meas. Technol..

[19] Huang H.Y., Yang C.H., Hsu W.H. A video watermarking algorithm based on pseudo 3D DCT. IEEE Symposium on Computational Intelligence for Image Processing.

[20] Li D., Cui L.H. (2017). Robust Animation Zero Watermarking Based on Visual Cryptography and Complete Complementary Code. Lect. Notes Electr. Eng..

[21] Liu X. (2020). Research on Digital Watermarking Algorithm for Audio and Video Matching. Master’s Thesis.

[22] Li D., Yang S., Zuo Y., Zheng Z., Cui L. (2018). Animation Zero Watermarking Algorithm Based on Edge Feature. Lecture Notes in Electrical Engineering.

[23] Cox I.J., Kilina J., Leighton T., Shamoon T. (1997). Secure spread spectrum watermarking for multimedia. IEEE Trans. Image Processing.

[24] Huang J.W., Shi Y.Q., Shi Q. (2000). Embedding Image Watermarks in DC Components. IEEE Trans. Circuits Syst. Video Technol..

[25] Lee D.D., Seung H.S. (1999). Learning the parts of objects with nonnegative matrix factorization. Nature.

[26] Medimegh N., Belaid S., Atri M., Werghi N. (2020). Statistical 3D watermarking algorithm using non-negative matrix factorization. Multimed. Tools App..

[27] Chen Z., Li L., Peng H., Liu Y., Yang Y. (2018). A novel digital watermarking based on General non-negative matrix factorization. IEEE Trans. Multimed..

[28] Da Cunha A.L., Zhou J., Do Minh N. (2006). The Nonsubsampled Contourlet Transform: Theory, Design, and Applications. IEEE Trans. Image Processing.

[29] Amiri A., Mirzakuchaki S. (2020). A digital watermarking method based on NSCT transform and hybrid evolutionary algorithms with neural networks. SN Appl. Sci..

[30] Narasimhulu C.V. A robust hybrid video watermarking algorithm using NSCT and SVD. Proceedings of the IEEE International Conference on Power, Control, Signals and Instrumentation Engineering (ICPCSI).

[31] Madenda S., Darmayantie A. (2021). Adaptive color space model based on dominant colors for image and video compression performance improvemen. Comput. Opt..

[32] Jiang Y., Cai M., Song C.H. (2018). Contourlet domain anti-attack video watermarking algorithm based on SIFT. Comput. Simul..

[33] Sang J., Liu Q., Song C.L. (2020). Robust video watermarking using a hybrid DCT-DWT approach. J. Electron. Sci. Technol..

